# Body Composition Profile of World-Class Male Water Polo Players in Relation to Position

**DOI:** 10.3390/jfmk11020243

**Published:** 2026-06-20

**Authors:** Milivoj Dopsaj, Athanasios A. Dalamitros, Klara Šiljeg, Andrea Perazzetti, Antonio Tessitore, Alexandros Nikolopoulos

**Affiliations:** 1 Faculty of Sport and Physical Education, University of Belgrade, 11030 Belgrade, Serbia; milivoj.dopsaj@fsfv.bg.ac.rs; 2 Department of Sport Sciences, Faculty of Sport and Health Sciences, Fit Generation Research Institute, AD500 Andorra la Vella, Andorra; dalammi@phed.auth.gr; 3 School of Physical Education and Sports Science, Aristotle University of Thessaloniki, 54124 Thessaloniki, Greece; 4 Faculty of Kinesiology, University of Zagreb, 10000 Zagreb, Croatia; klara.siljeg@kif.unizg.hr; 5 Degree Course in Sciences of Motor Activities, Sports and Psychomotor Education, Faculty of Law, Telematic University Giustino Fortunato, 82100 Benevento, Italy; a.perazzetti@unifortunato.eu; 6 Department of Movement, Human and Health Sciences, University of Rome ‘Foro Italico’, 00135 Rome, Italy; antonio.tessitore@uniroma4.it; 7 School of Physical Education & Sport Sciences, National and Kapodistrian, University of Athens, 11527 Athens, Greece

**Keywords:** morphological characteristics, body composition, playing position, water polo

## Abstract

**Background and Objectives**: Water polo (WP) is a high-intensity, intermittent aquatic team sport that has been extensively investigated within sports science. While contemporary literature has examined the body composition and morphological characteristics of elite and international WP players, this study aimed to define the general body composition profile of world-class WP players and determine position-specific differences. **Methods**: The study involved 72 national team players from Serbia, Croatia, Greece, and Italy who participated in the Olympic Games, World Championships, or European Championships. Participants’ body composition was measured using the InBody 720 multichannel bioimpedance method. Ten different variables were examined to assess body structure regarding contractile and ballast components. **Results**: MANOVA revealed statistically significant differences in body composition across playing positions (Wilks’ lambda = 0.239, *p* < 0.000, η^2^_p_ = 0.402). The variables that had the greatest impact on the difference were: body mass, body fat and body mass index with the 47.0, 44.4, and 43.7% of explained total variance of the impact on the differences (*p* = 0.000), respectively. **Conclusions**: world-class WP players assigned to different playing positions differ significantly in body composition. These positional profiles should be considered in talent identification, selection procedures, training, and nutritional strategies to optimize performance models, considering the future evolution of the game at the highest competitive level. Coaches could use this information to initially select players for different specific positions based on anthropometric and body composition criteria.

## 1. Introduction

Research on anthropometric body characteristics and body composition in athletes of different sports has proven the existence of great specificities in the function of sport, as well as the position of the game, because these characteristics positively contribute to the part of explanation of sports performance and competitive success [1,2]. The importance of monitoring and controlling body status, longitudinally during an athlete’s development and career, and in terms of evolution of particular sport, is reflected in the fact of a direct connection between the physical abilities and body composition, where muscle mass positively influences performance in activities that require muscular strength, explosiveness, anaerobic speed or agility, while excess fat negatively affects different types of endurance and capacity [3,4,5,6]. However, unlike most land sports, where body fat is negatively related to performance, body fat can be very beneficial in water polo as a hydrodynamic advantage. The fact is that fat is less dense than muscle or water, which helps the body float, which can help players stay afloat with less energy [7].

Water polo (WP) is one of the oldest disciplines in the Olympic Games program and, along with other water sports, is one of the team sports that takes place exclusively in a water environment. Water polo is widely recognized as a physically, metabolically, tactically, and cognitively demanding intermittent team sport [8,9]. From a physiological perspective, it is characterized by repeated moderate- to high-intensity swimming sprints performed under direct physical opposition, interspersed with short periods of lower-intensity activity or highly intense vertical (eggbeater) and grappling actions. Heart rate responses recorded during competition, together with elevated blood lactate concentrations, indicate that players operate predominantly within high and submaximal aerobic zones, with frequent transitions into high-intensity anaerobic metabolism [10,11,12]. In terms of game organization, one team plays with seven players, six of whom are field players and a goalkeeper. For men, the field area was 30 × 20 m by 2025, but it was reduced to 25 × 20 m under new rules that apply from 2026, with a water depth of at least 2 m. The playing field in WP has an area of 500 m^2^, while one player should theoretically cover an area of 41.67 m^2^ with his activity.

Over recent decades, WP has been extensively investigated within sports science. Research has addressed players’ physical capacities [11,12], the effectiveness of specific training methodologies [13,14], and physiological, health-related, and injury-related aspects [3,10,15,16]. Considerable attention has also been devoted to the development of body composition models [4,7,16,17,18], as well as to technical-tactical performance analysis and competitive match characteristics [5,8,19,20]. Additional studies have examined age-group categories and gender-specific considerations [21,22,23,24]. Collectively, these reports underscore the substantial scientific interest in the multidimensional phenomenology of WP.

Although extensively studied, most of the previous work has analyzed body composition in WP players focused on age-group player selections or individual national teams [3,4,7,16,21,25,26]. As WP continues to evolve globally and undergoes regulatory modifications, there is a clear need to establish an international reference model or standardized profile of body composition for elite athletes [23].

Currently, comprehensive data on world-class WP players, considered as elite performers, remain limited, particularly regarding generalized body composition profiles and position-specific morphological differentiation. Addressing this gap constitutes the primary aim of the present research. The practical objective of this study is to provide statistically grounded sports metrology evidence that may support future evaluation of morphological characteristics in WP players. Specifically, the findings are expected to contribute to talent identification and selection processes, optimization of training adaptations (both traditional and strength-conditioning programs), and the refinement of nutritional strategies, dietary models, and supplementation protocols tailored to positional demands at the highest competitive level.

## 2. Materials and Methods

### 2.1. Study Design

The research was implemented as a cross-sectional cohort study [27]. The subjects were measured using laboratory methods successively in the period from 2018 to 2023 according to the principle of a multicenter study. This research was conducted in accordance with the Declaration of Helsinki for recommendations of physicians in biomedical research involving humans [28] and was approved by the Ethics Committee of the Faculty of Sport and Physical Education, University of Belgrade (approval number 484-2). All players, WP clubs or national teams, were informed of the purpose and objectives of the study and gave their informed consent.

### 2.2. Sample

The study involved 72 top WP players from Serbia (35), Croatia (14), Greece (10) and Italy (13). All players (age = 27.8 ± 4.7 years., Min-Max = 18.4–37.0 years.; training experience = 19.5 ± 4.5 years., Min-Max = 9.0–28.5 years.) were members of official national teams that participated in the Olympic Games (OG), World (WC) or European Championships (EC) in the given period. All four teams won a total of 11 medals at the aforementioned period of time and competitions, and players surveyed in this study played for their national team in the year they won the medal. So it can be considered that the players in the sample represent the world-class WP players. World-class athletes (Tier 5) are also defined as athletes who are an Olympic and/or world medalists, or athletes achieving world-leading performance or top players within top teams (teams which have a medal or are in the most competitive leagues) [29]. The total sample of players was divided into subsamples according to specific playing positions in the game: Goalkeepers (G, *n* = 11), central guards (CG, *n* = 17), central players (CP, *n* = 8), and outside players (OP, *n* = 36).

### 2.3. Body Composition Measurement

Written informed consent was obtained from the participating coaches and athletes prior to the commencement of the study. All measurements were performed at the Faculty of Sports and Physical Education, University of Belgrade, in the Methodological Research Laboratory by the same staff on the same equipment (InBody 720/InBody, Seoul, Republic of Korea), which was regularly maintained and calibrated by the official technical service (https://borf.rs/, available at 15 March 2026). The InBody 720 is a respected multi-frequency BIA tool, acknowledging that BIA was used by sports and nutritional scientists as a valuable laboratory and field-testing technology [3,6,30]. All measurements were carried out in accordance with an implemented protocol previously applied [30,31,32]. Players are asked to adhere to the following pre-test guidelines 24 h prior to the measurement: they were asked to refrain from consuming food or large amounts of drinks for at least 4 h before, and not to drink coffee for at least 12 h before the measurement; before the measurement, they were recommended to go to the toilet (according to personal needs), while they were prohibited from sitting or lying down for a minimum of 2 min immediately before the measurement; an agreement was made with the coaches that the players would not have a strong training session the day before the measurement, but only a light training session; during the measurement procedure, the players were only in their underwear without any metal jewelry on them. All individual player measurements during the given research period were always carried out in the morning hours from 08:00 to 09:00, in a standardized environmental condition in the lab.

### 2.4. Variables

All examined variables were defined based on the body composition profile of WP players from the aspect of basic anthropo-morphological measures, as well as the determination of contractile and ballast related body components, i.e., metrics of the voluminosity and longitudinally of the body [6,30]. Also, two index variables were quantified, characterized as important informative indicators of the body composition structure of the participants [16,31,32,33]. The following variables were analyzed:

1. body height (BH), as a measure of body longitudinally, expressed in cm;
2. body mass (BM), as a measure of body voluminosity, expressed in kg;
3. body mass index (BMI), calculated as: BM/BH^2^ (m), expressed in kg·m^−2^;
4. skeletal muscle mass (SMM), as an amount of skeletal muscles as a contractile tissue mass in the body, expressed in kg;
5. percentage of skeletal muscle mass (PSMM), as a measure of SMM standardized by body voluminosity, calculated as: SMM/BM, expressed in %;
6. skeletal muscle mass index (SMMI), as a measure of the muscle mass of the body standardized by cross section of body longitudinally, calculated as: SMM/BH^2^ (m), expressed in kg·m^−2^;
7. body fat mass (BFM), as an amount of fat mass as a ballast tissue mass in the body, expressed in kg;
8. percentage of body fat (PBF), as a measure of body fat mass standardized by voluminosity, calculated as: BF/BM, expressed in %;
9. fat mass index (FMI), as a measure of body fat mass standardized by cross section of body longitudinally, calculated as: FM/BH^2^ (m), expressed in kg·m^−2^;
10. muscle-fat index (MFI), calculated as relation between SMM (in kg) and BFM (in kg), expressed in kg;
11. index of body composition (IBC), calculated as relation between BMI (in kg·m^−2^) and PBF (in %), expressed in Index Unit (IU).

### 2.5. Statistical Analyses

All raw data were analyzed using descriptive statistics (mean value—Mean, standard deviation—SD, coefficient of variation—cV%, lower and upper bound 95% Confidence Interval for Mean, and minimal and maximal measured variables values—Min, Max). The normality of the distribution of variables was calculated by applying the non-parametric Kolmogorov–Smirnov test (KS Z). For variables that did not have a normal distribution, the Box–Cox transformation was used to transform the raw data into a form much closer to a normal distribution, and then the general difference between the groups was determined by MANOVA. One-way ANOVA was used to determine differences between groups in terms of individual variables, using Welch’s assumption of inequality of variance. The Games-Howell post hoc test criterion was used to calculate differences between pairs of variables. The effect of size was calculated by partial eta square (η^2^_p_) [34,35].

The standardized values of the differences were calculated using the Z-score method, in relation to the general model, i.e., the mean of all examined players, and were expressed in percentages (%), while the summarized standardized differences values were calculated based on following formula: for contractile component as mean value of SMM, PSMM and SMMI; for ballast component as Mean of BF, PBF and BFMI; and for nutritional status component as mean of BMI, MFI and IBC. Statistical significance [34] was set at criteria 0.05 (*p* ≤ 0.05), while IBM SPSS 25.0 statistical software (IBM Corp., Armonk, NY, USA), and Jamovi 2.6.44 (https://www.jamovi.org/, available at 22 March 2026) was used for all statistical calculations.

## 3. Results

Table 1 shows all the results of descriptive statistics, with the results of the test of normality of the distribution of the examined variables as a general model of world-class level male WP players.

The results of MANOVA showed that between the players divided by positions as a function of the investigated body composition variables, there is a general statistically significant difference at the level of Wilks’ lambda value = 0.239, F = 3.26, *p* = 0.000, partial eta squared (η^2^_p_) = 0.402, observed power = 1.000.

Table 2 shows all the results of descriptive statistics according to playing position, with the results of the ANOVA and between position differences in the examined variables.

Table 3 shows the values of standardized differences in the examined variables of the mean values of the studied sample of water polo players in relation to the mean values of the players according to the positions in the game.

**Figure 1:**
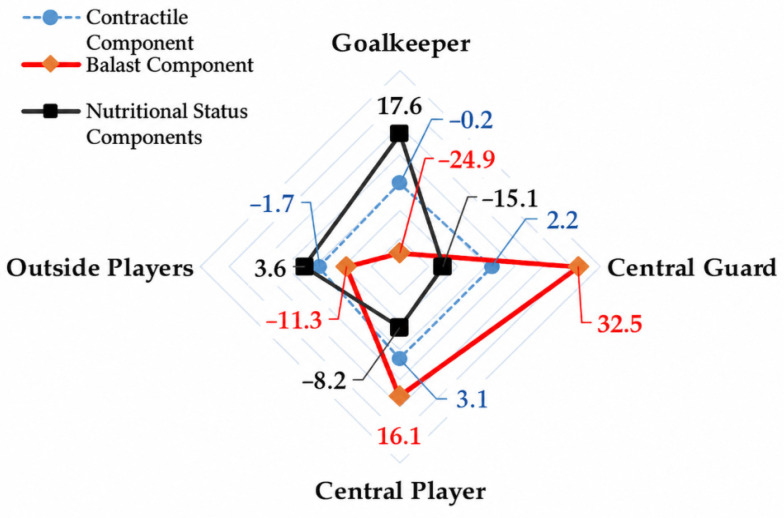
Radar chart of summarized standardized differences (expressed in %) of the examined structured body composition variables according to playing position.

Figure 1 shows the results of the average standardized score differences between playing positions in relation to different structural aspects of the players’ body composition (contractile component is calculated as the average of the values of the individual variable score differences in the total sample in relation to the strata of the playing position sample for the variables SMM, PSMM and SMMI; ballast component is calculated in the same way for BF, PBF and BFMI; nutritional status component is calculated in the same way for the variables BMI, MFI and IBC).

## 4. Discussion

The present study aimed to define the body composition profile of world-class WP players (Tier 5) and to examine position-specific differences. The results confirmed significant multivariate differences across playing positions, supporting the study hypothesis and reinforcing the importance of position-dependent morphological specialization in male elite WP players.

Elite water polo athletes, over the course of their long-term training careers, are exposed to systematic and high-volume training programs, particularly during pre-competitive and competitive phases [12]. Optimal performance at the elite level is strongly associated with complex physiological and morphological adaptations and represents the outcome of multiple interacting factors. These include appropriate training structure in terms of volume and intensity, effective integration of concurrent training modalities (gym and pool training), optimized nutritional strategies and supplementation protocols, structured recovery models, and continuous health monitoring aimed at preventing acute and chronic overtraining or injury [12,13].

At the global level, longitudinal analyses of morphological development over the past century indicate that athletes in many sports have progressively become taller and more massive, with growth rates exceeding the secular trend observed in the general population [1]. In open-category sports without weight classifications, body size and structural characteristics often represent critical determinants of performance potential. For example, in basketball and volleyball, specific anthropometric attributes are strong predictors of selection and long-term success, highlighting the importance of morphological profiling in talent identification processes [2,30].

### 4.1. General Morphological Profile

The results showed that the basic body characteristics of world-class WP players are as follows: BH = 192.7 kg, BM = 97.7 kg, and BMI = 26.31 kg·m^−2^, respectively (Table 1). General and partial statistically significant differences were found between the basic body variables in relation to the position in the game at the level of *p* = 0.000 (Table 2). The largest variance of differences was found in BM (η^2^_p_ = 0.470, 47.0%) and it can be concluded that body mass (BM) is the basic body variable that dominantly distinguishes the body measure of world-class WP players in relation to positions.

Official data on the BH adult population show that the average height of men in Serbia is 180.9 cm, in Croatia 180.5 cm, in Greece 178.1 cm and in Italy 176.5 cm, i.e., on average 179.0 cm [36], which leads to the conclusion that world-class WP players, in general, belong to the stratum of above-average tall individuals compared to the general population (taller by 13.7 cm, or 7.65%).

In relation to relevant data on the height of WP players from other National or Olympic teams, it was established that the members of the Italian male team that participated in the 2016 OG were 190.0 ± 5.54 cm high [26], the players from the participating teams of OG 2021 Serbia, USA and Montenegro were on average 191.02 ± 4.95, 191.49 ± 5.93 and 191.79 ± 4.76 cm high, respectively [17], while top Hungarian players are between 193.5 ± 5.8 cm and 194.6 ± 4.9 cm in height [3,37]. The players from the Iranian Olympic water polo team were 186.5 ± 2.0 cm tall [16]. According to data on the anthropo-morphological status of the elite top club level Croatian WP players in 1980 and 1995, they were 185.9 and 189.6 cm tall, respectively, meaning that body high between two different generations of elite national male WP players 15 years apart was different 3.7 cm or 1.99% [7]. Results of this research showed that modern water polo players are on average 192.7 cm tall, which suggests that the average height of elite players has been continuously increasing over the last 45 years, apparently most probably due to the selection and the needs of the modern water polo game.

Based on the results of the average values of PSMM and PBF (Table 1, 49.60 ± 2.46 and 13.68 ± 4.17%, respectively), it can be concluded that, in general, world-class WP players belong to the category of athletes with a high percentage of muscles in the body, and a low percentage of body fat, which is in accordance with data on top athletes, athletes from sports games, and in accordance with previous data on top water polo players [1,3,7,18,26,30]. In relation to MFI, there is currently no adequate data in the available literature for comparison, while in relation to IBC, the average value from the studied sample is twice as high as in the adult healthy male population and that it indicates a body status with a high capacity for producing high values of maximal muscle force and maximal muscle explosiveness [31]. It should also be emphasized that only these variables (MFI and IBC, Table 1) did not have a normal distribution, which indicates the extreme dispersion of the given indices, so in future research it is necessary to determine their sports-ecological validity.

### 4.2. Position-Specific Adaptations

In terms of playing positions, G are the tallest (197.4 cm), then CG (195.2 cm), CP (193.6 cm), and the shortest are OP (189.9 cm). In terms of height, players differ significantly, where G and CG were significantly taller than OP (Table 2.)

As with the general population, it has been established that athletes in many sports become taller and more massive over time, with even elite athletes having growth rates that exceed those of the general population secular trend. In open-type sports, i.e., sports without a defined weight category, it is obvious that taller and larger players have an advantage [1]. Definitively, the biggest players in WP are the CG and CP, that is, players who play in the position with the most intense duels, and in the position directly in front of the opponent goal.

In relation to BM and BMI, as measures of mass and overall volume of the body, it can be argued that there are clearly defined two body type groups of players. Statistically, the most massive and largest are CG and CP (BM = 108.4 vs. 105.5 kg, and BMI = 28.43 vs. 28.16 kg·m^−2^, respectively), compared to G and OP (BM = 94.7 vs. 91.9 kg, and BMI = 24.33 vs. 25.50 kg·m^−2^, respectively, Table 2). Compared to standardized difference values CG and CP at average are almost 9.5% heavier and they have 7.6% more body volume than goalkeepers and outside players, respectively (Table 3). It has previously been found that in the case of Spanish elite players, central strikers have significant anthropometric differences compared to other specific playing positions. The authors concluded that these data reflect the importance of muscle mass, especially due to the need for greater upper body strength in relation to speed and throwing skills [38].

It means that in modern WP, from the aspect of body status and body composition, generally speaking, selection, gym training and diet must be adapted to the needs of the position in the game. Also, coaches can use this information in the process of selecting players for different specific positions.

Interestingly, the results showed that the current fact that world-class CG position players have a level of ballast component (fat) in their body composition, 32.5% higher than the standardized model of world-class WP players (Figure 1). One possible explanation is that the primary task of the CG is to guard and prevent the opponent’s CP from receiving the ball. This means that during the defensive phase of the game, the CG must always be dominantly in a higher floating position directly next to the opponent’s CP, with a tendency to push him out of the danger line for a possible scoring goal. It is likely that the biophysical advantage, as well as the buoyancy factor, favors CG, to have a massive body and higher body fat percentage, which may help a CG stay high in the water during intensive duels with CP, making eggbeater kicking more energetically efficient, especially in the hands-up position. In addition to specific WP skills, a technical-tactical (TE-TA) advantage of a team is to have a specifically skilled, larger and more physically buoyant player on the first line of defense, i.e., directly in front of the goal [4,9,12].

On the other hand, the highest standardized level of contractile component by body height was established in players in the CP position, where they had the highest values of SMMI (13.52 kg·m^−2^, the highest amount of SMM per one m^2^ cross section of body, 5.1% more than the standardized value, Table 2 and Table 3, respectively). It has previously been established that in top-level WP, such as the Euroleague or the Italian Serie A1 and Serie A2, the dominant tactical option of attacking play is performing a high occurrence of Power-Play possessions following an exclusion, which is especially the case with central players during possession with a player more [39], or during the typical CP position play during the regular attacking as a dominant part of the game [13]. This tactical concept of Power Play requires the players to be prepared for the realization of highly intensive and rapid movement anaerobic and aerobic efforts [8,12,38]. It was found that lactate values during a match vary between positions, with the CP position having significantly higher average blood lactate concentrations (11.2 mmol/L^−1^) than the CG (6.7 mmol/L^−1^) and peripheral position (5.3 mmol/L^−1^) [40]. It is most likely that the structure of competitive loads CP have to perform during a match is a consequence of the specific body composition adaptation towards to dominant muscle mass level and high intensive moment of play and fast movement performance in relation to other positions [41,42,43].

However, in relation to relative measures of the amount of contractile component in the body, the highest values were found in G and OP (PSMM = 51.33 and 50.23%, respectively, Table 2). Also, the ratio between contractile and ballast tissue in the body is highest in G and OP, which means that they on average have 5.84 and 4.62 times more muscle (contractile) than ballast (fat) tissue (Table 2).

The goalie’s task is to defend the goal area from opponent shots, where the fastest possible reactions and rapid movements, both single and consecutive, are crucial to attempting to execute a given defense effectively. This is probably why goalkeepers are the tallest players (197.4 kg), players with the highest relative percentage of muscle in the body (51.33%), the lowest percentage of body fat (10.64%), and the highest ratio of muscle to fat component in the body (5.84 kg). Namely, fast reactions, pronounced longitudinally and emphasized leanness and muscularity are the dominant physical characteristics of world-class goalkeepers (Table 2).

From a practical standpoint, players in the CG and CP position have the absolute largest muscle mass and fat mass of all players, and this can be seen as a phenomenology of dominant physical body adaptation in relation to the opponent in the game (CG) and the consequent duel TE-TA task, while in players in the G and PP positions, the phenomenology of physical adaptation of body composition was dominantly carried out in relation to the relative part of the muscle mass in the body, based on the requirements of the court space and the consequent TE-TA task of the game.

### 4.3. Limitations of the Study

A major limitation of the study is the fact that the researchers were unable to control the subjects’ diet, hydration status, and training effects the day before testing, despite clearly provided instructions and the players’ consent to the testing conditions. Also, during the time period of data collection for the study, the measuring instrument (BIA InBody 720) was regularly maintained and calibrated by an official technical service, so we believe that any measurement errors that may arise due to measurement inconsistencies were completely minimized.

## 5. Conclusions

Based on these results, it can be concluded that world-class WP players assigned to different playing positions differ statistically significantly in terms of body composition. Also, based on the determined characteristics of the players’ body composition it can be hypothetically assumed that the training and competitive adaptations, as well as the specific selection of players with CG and CP, are predominantly carried out in relation to the characteristics of the opponent, while in G and PP they are carried out in relation to the TE-TA requirements of the game in the field.

## Figures and Tables

**Table 1:** Descriptive statistics and normality test of the distribution of the examined variables as a general model of world-class level male WP players.

Variables	Mean ± SD	cV%	95% Confidence Interval for Mean		Min	Max	Test of Normality	
			Lower Bound	Upper Bound			KS Z	*p*
BH (cm)	192.7 ± 6.1	3.17	191.3	194.1	179.3	204.5	0.081	0.120
BM (kg)	97.7 ± 10.4	10.69	95.3	100.2	74.2	120.8	0.059	0.926
BMI (kg·m^−2^)	26.31 ± 2.34	9.32	25.76	26.86	20.86	29.79	0.084	0.665
SMM (kg)	48.36 ± 4.61	9.26	47.27	49.44	38.70	57.60	0.098	0.490
PSMM (%)	49.60 ± 2.46	5.21	49.01	50.17	44.90	54.99	0.075	0.790
SMMI (kg·m^−2^)	13.01 ± 0.83	6.48	12.81	13.20	11.37	14.48	0.071	0.837
BF (kg)	13.60 ± 4.91	37.82	12.45	14.75	3.20	23.30	0.084	0.690
PBF (%)	13.68 ± 4.17	31.96	12.70	14.67	3.85	21.67	0.064	0.907
BFMI (kg·m^−2^)	3.68 ± 1.33	38.54	3.36	3.99	0.80	6.41	0.049	0.991
MFI (kg)	4.25 ± 2.38	55.79	3.69	4.81	2.07	14.28	0.210	0.003
IBC (IU)	2.14 ± 0.83	38.39	1.95	2.33	1.36	5.42	0.182	0.015

Note: BH = body height; BM = body mass; BMI = body mass index; SMM = skeletal muscle mass; PSMM = percent of skeletal muscle mass; SMMI = skeletal muscle mass index; BF = body fat; PBF = percent of body fat; BFMI = Body Fat Mass Index; MFI = muscle-fat index; IBC = index of body composition.

**Table 2:** Descriptive statistics according to playing position.

Variables	Position	MEAN ± SD	cV%	95% Conf. Interv. for Mean		ANOVA and Partial Eta^2^	Between Positions Differences
				Low. Bound	Upp. Bound		
BH (cm)	G	197.4 ± 3.8	2.49	194.8	199.9	F = 7.28; *p* = 0.000; η^2^_p_ = 0.243	G vs. OP *p* = 0.001; CG vs. OP *p* = 0.009
	CG	195.2 ± 4.9	2.36	192.7	197.7		
	CP	193.6 ± 3.4	1.76	190.8	196.5		
	OP	189.9 ± 6.3	3.37	187.8	192.0		
BM (kg)	G	94.7 ± 8.9	9.12	88.7	100.7	F = 20.1; *p* = 0.000; η^2^_p_ = 0.470	G vs. CG *p* = 0.002; G vs. CP *p* = 0.011; CG vs. OP *p* = 0.000; CP vs. OP *p* = 0.000
	CG	108.4 ± 7.2	6.72	104.7	112.1		
	CP	105.5 ± 2.3	2.18	103.6	107.4		
	OP	91.9 ± 8.5	9.57	89.1	94.8		
BMI (kg·m^−2^)	G	24.33 ± 2.40	9.56	22.72	25.94	F = 17.6; *p* = 0.000; η^2^_p_ = 0.437	G vs. CG *p* = 0.000; G vs. CP *p* = 0.001; CG vs. OP *p* = 0.000; CP vs. OP *p* = 0.001
	CG	28.43 ± 1.15	4.39	27.84	29.02		
	CP	28.16 ± 0.83	2.95	27.47	28.85		
	OP	25.50 ± 1.97	9.15	24.83	26.17		
SMM (kg)	G	48.50 ± 3.89	8.83	45.88	51.12	F = 8.71; *p* = 0.000; η^2^_p_ = 0.278	CG vs. OP *p* = 0.000; CP vs. OP *p* = 0.003
	CG	51.55 ± 3.67	6.45	49.66	53.44		
	CP	51.31 ± 2.74	5.34	49.02	53.60		
	OP	46.15 ± 4.38	9.16	44.67	47.63		
PSMM (%)	G	51.33 ± 2.36	5.28	49.74	52.92	F = 9.22; *p* = 0.000; η^2^_p_ = 0.289	G vs. CG *p* = 0.002; CG vs. OP *p* = 0.000
	CG	47.59 ± 1.80	3.65	46.66	48.51		
	CP	48.62 ± 2.24	4.61	46.75	50.49		
	OP	50.23 ± 2.16	4.91	49.51	50.96		
SMMI (kg·m^−2^)	G	12.45 ± 0.95	7.33	11.82	13.89	F = 8.43; *p* = 0.001; η^2^_p_ = 0.289	G vs. CG *p* = 0.020; G vs. CP *p* = 0.015; CG vs. OP *p* = 0.002; CP vs. OP *p* = 0.014
	CG	13.52 ± 0.57	4.09	13.23	13.82		
	CP	13.68 ± 0.60	4.39	13.18	14.18		
	OP	12.78 ± 0.74	5.95	12.53	13.03		
BF (kg)	G	10.26 ± 4.27	41.59	7.39	13.14	F = 18.20; *p* = 0.000; η^2^_p_ = 0.445	G vs. CG *p* = 0.000; G vs. CP *p* = 0.024; CG vs. OP *p* = 0.000; CP vs. OP *p* = 0.040
	CG	18.73 ± 3.70	20.51	16.83	20.63		
	CP	16.15 ± 3.64	22.54	13.10	19.20		
	OP	11.63 ± 3.61	38.43	10.41	12.85		
PBF (%)	G	10.64 ± 3.92	39.05	8.01	13.28	F = 10.20; *p* = 0.000; η^2^_p_ = 0.310	G vs. CG *p* = 0.000; G vs. OP *p* = 0.000;
	CG	17.24 ± 3.04	17.45	15.68	18.81		
	CP	15.32 ± 3.49	22.78	12.40	18.23		
	OP	12.57 ± 3.65	33.44	11.34	13.81		
BFMI (kg·m^−2^)	G	2.65 ± 1.14	44.72	1.88	3.42	F = 13.90; *p* = 0.000; η^2^_p_ = 0.380	G vs. CG *p* = 0.000; G vs. CP *p* = 0.021; CG vs. OP *p* = 0.000;
	CG	4.92 ± 0.97	20.16	4.42	5.42		
	CP	4.33 ± 1.04	24.02	3.45	5.20		
	OP	3.26 ± 1.09	41.74	2.89	3.62		
MFI (kg)	G	5.84 ± 3.33	59.71	3.61	8.08	F = 4.94; *p* = 0.004; η^2^_p_ = 0.179	CG vs. OP *p* = 0.001
	CG	2.87 ± 0.64	21.53	2.54	3.20		
	CP	3.35 ± 0.88	26.27	2.61	4.08		
	OP	4.62 ± 2.43	52.48	3.80	5.44		
IBC (IU)	G	2.63 ± 1.13	44.84	1.87	3.39	F = 3.72; *p* = 0.015; η^2^_p_ = 0.141	CG vs. OP *p* = 0.007
	CG	1.69 ± 0.29	16.01	1.55	1.84		
	CP	1.91 ± 0.39	20.57	1.59	2.24		
	OP	2.25 ± 0.87	38.24	1.96	2.55		

Note: Goalkeeper = G; central guard = CG; central player = CP; outside player = OP; BH = body height; BM = body mass; BMI = body mass index; SMM = skeletal muscle mass; PSMM = percent of skeletal muscle mass; SMMI = skeletal muscle mass index; BF = body fat; PBF = percent of body fat; BFMI = Body Fat Mass Index; MFI = muscle-fat index; IBC = index of body composition.

**Table 3:** Standardized differences (expressed in %) of the examined body composition variables in players according by playing position.

Variables	Goalkeeper	Central Guard	Central Player	Outside Players
BH (cm)	2.4	1.3	0.5	−1.5
BM (kg)	−3.1	11.0	8.0	−5.9
BMI (kg·m^−2^)	−7.5	8.1	7.0	−3.1
SMM (kg)	0.3	6.6	6.1	−4.6
PSMM (%)	3.5	−4.1	−2.0	1.3
SMMI (kg·m^−2^)	−4.3	3.9	5.1	−1.8
BF (kg)	−24.6	37.7	18.8	−14.5
PBF (%)	−22.2	26.0	12.0	−8.1
BFMI (kg·m^−2^)	−28.0	33.7	17.7	−11.4
MFI (kg)	37.4	−32.5	−21.2	8.7
IBC (IU)	22.9	−20.8	−10.5	5.1

Note: BH = body height; BM = body mass; BMI = body mass index; SMM = skeletal muscle mass; PSMM = percent of skeletal muscle mass; SMMI = skeletal muscle mass index; BF = body fat; PBF = percent of body fat; BFMI = Body Fat Mass Index; MFI = muscle-fat index; IBC = index of body composition.

## Data Availability

The research data used in the analysis will be available upon request, through the first or corresponding author.

## Abbreviations

The following abbreviations are used in this manuscript:

WP	Water Polo
BH	Body Height
BM	Body Mass
BMI	Body Mass Index
SMM	Skeletal Muscle Mass
PSMM	Percent of Skeletal Muscle Mass
BF	Body Fat
PBF	Percent of Body Fat
BFMI	Body Fat Mass Index
MFI	Muscle Fat Index
IBC	Index of Body Composition
G	Goalkeepers
CG	Central Guards
CP	Central Players
OP	Outside Players
